# Frailty as a predictor of hospital length of stay after elective total joint replacements in elderly patients

**DOI:** 10.1186/s12891-018-1935-8

**Published:** 2018-01-16

**Authors:** Han Ting Wang, Josée Fafard, Stéphane Ahern, Pascal-André Vendittoli, Paul Hebert

**Affiliations:** 1 Department of Internal medicine and Critical Care Medicine, Centre Integre Universitaire de Sante et Services Sociaux (CIUSSS) de l’est de l’île de Montréal, Hopital Maisonneuve-Rosemont, 5415 boul. l’Assomption, H1T 2M4, Montreal, Quebec, Canada; 2 Department of Surgery, Centre Integre Universitaire de Sante et Services Sociaux (CIUSSS) de l’est de l’île de Montréal, Hôpital Maisonneuve-Rosemont, 5415 boul. l’Assomption, H1T 2M4, Montreal, Quebec, Canada; 3 Departments of Medicine and Critical Care Medicine and Centre de recherche, Centre hospitalier de l’Université de Montréal (CRCHUM), 900 St-Denis, H2X 0A9, Montréal, Québec, Canada

**Keywords:** Frailty, Elderly, Total joint replacement, Outcome, Knee arthroplasty, Hip arthroplasty

## Abstract

**Background:**

Total joint replacement procedures are increasing in number because of population aging and osteoarthritis development. Defined as a lack of physiological reserves and the inability to adequately respond to external stressors, frailty may be more common than expected in older patients with degenerative arthritis awaiting total joint replacements.

The aim of the present study was to assess associations between frailty and adverse outcomes, frailty prevalence among elderly patients awaiting elective TJR, and agreement between 2 frailty screening instruments.

**Methods:**

We undertook a prospective, observational, pilot study in our institution. We enrolled patients 65 years or older who were awaiting elective knee or hip replacement surgery and evaluated them in our preoperative clinic with planned postoperative hospital length of stay greater than 24 h. Patients were asked to grade their perceived well-being on the Clinical Frailty Scale and to answer questions on the FRAIL Scale.

**Results:**

The Clinical Frailty Scale classified 40 patients (45.9%) as robust, 43 patients (49.4%) as prefrail and 4 patients (4.5%) as frail, while the FRAIL Scale categorized 12 patients (13.7%) as robust, 54 patients (62.0%) as prefrail, and 20 patients (22.9%) as frail. Robustness, ascertained on the Clinical Frailty Scale was, while the FRAIL Scale was not, significantly associated with shorter hospital length of stay and fewer discharges to the rehabilitation center. Both scales showed moderate mutual agreement.

**Conclusion:**

Screening for frailty identified between 5% and 10% of patients at risk of adverse outcomes. The Clinical Frailty Scale was, while the FRAIL scale was not, significantly associated with hospital length of stay and discharge to rehabilitation center in our cohort of total joint replacement patients.

## Background

Total joint replacement (TJR) procedures are increasing in number with population aging and osteoarthritis development. In its 2014 annual report, the Canadian Joint Replacement Registry recorded a 16.5% surge of hip replacements and a 21.5% proliferation of knee replacements in the last 5 years [1] paralleled by increased frailty with advancing age in the general population [2]. Frailty embodies insufficient physiological reserves and the inability to adequately respond to external stressors. Its etiology is complex but is related to aging, faster senescence and acquired illnesses [3, 4]. Its pervasiveness in community-dwelling elders is estimated to be approximately 10%, depending on the study cited [5], and is associated with increased 1-year mortality and institutionalization.

Frailty prevalence is generally considered to be even greater in surgical pre-operative settings, varying from 10% to 46%, depending on the screening instrument selected, the surgical subspecialty/setting, and patient characteristics [6–8]. The choice of “frailty scale” may not be obvious owing to the variety of available instruments with different conceptual underpinnings [9, 10]. Nonetheless, in most studies, frailty is a better predictor of post-operative outcomes than co-morbidity scores and even some validated risk model scores specific to certain procedures [6–8].

In orthopedic surgery, especially in patients awaiting TJR, few teams have looked at the association between frailty and post-operative outcomes, such as hospital length of stay (LOS) and disposition at discharge. Frailty may help in selecting vulnerable patients when combined with known predictors in TJR procedures (i.e., pre-operative hemoglobin (Hb) level, American Society of Anesthesiologists score, nutritional status, etc.) [11–13]. The lack of consensus on the different available models of frailty makes it challenging to choose appropriate screening instruments.

Therefore, we undertook a study, with 2 frailty-screening instruments, to assess associations between frailty, hospital LOS and transfer to rehabilitation centre or not among elderly patients awaiting elective, primary TJR. We hypothesized that frail patients are less likely to be discharged home and will have longer LOS. As secondary objectives, we compared the prevalence of frailty with 2 instruments and evaluated their agreement.

## Methods

### Setting

We conducted a prospective, observational study in our tertiary academic hospital with a pre-operative clinic that sees over 3000 patients per year, and approximatively 20% of them are orthopedics-related.

### Study participants

**Fig. 1:**
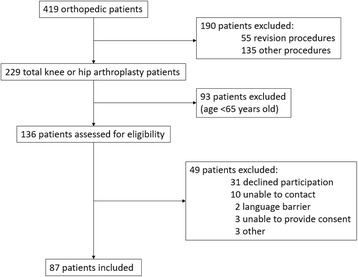
Patient recruitment flow chart

From November 2015 to May 2016, we identified orthopedic participants through routine daily screening of our pre-operative clinic schedule (*N* = 419). A research nurse was trained for screening purposes and performed all frailty assessments. Patients 65 years or older and awaiting primary TJR were enrolled and evaluated in our pre-operative clinic with planned post-operative hospital stay greater than 24 h. For feasibility reasons, we excluded all urgent procedures, patients deemed unfit for surgery by their surgeon or medical consultant, and those unable to understand or provide consent. If they fulfilled eligibility criteria, patients were contacted by phone or in person, and informed consent was obtained. Those who gave consent were subsequently interviewed for frailty assessment. Figure 1 shows the number of patients meeting inclusion and exclusion criteria. In summary, a total of 87 patients accepted to participate and completed the study. Full approval by our local Research Ethics Committee was granted before the study was started.

### Frailty assessment

We chose to define frailty as an age-associated, multi-dimensional syndrome of insufficient reserves giving rise to vulnerability [14]. Based on the current literature [9], the 2 most commonly-used frailty models in surgical settings are Rockwood’s deficit accumulation model [15] and Fried’s phenotypic model [16]. Rockwood’s model was quantified with the Clinical Frailty Scale, and Fried’s model was assessed with the FRAIL Scale. The Clinical Frailty Scale, derived from the Frailty Index, is an ordinal scale from 1 to 8, where 1 represents a state of robustness, and 8 indicates a very severe frailty state. Patients were asked to identify which level on the scale best corresponded to their perceived well-being. They can be further grouped as being robust (score 1 to 3), pre-frail (score 4), and frail (score 5 to 8), based on the initial study by Rockwood et al. [17]. It is well-validated in predicting adverse outcomes in community-dwelling elders [15].

The Clinical Frailty Scale was selected over the Frailty Index as being more feasible in clinical practice. The FRAIL Scale was created at a consensus meeting of the International Academy on Nutrition and Aging Task Force. Its conceptual underpinnings are heavily rooted in Fried’s phenotypic model [18]. It is composed of 5 items (fatigue, resistance, ambulation, weight loss, illness) with the first 4 components taken from Fried’s phenotypic model. Also, it is assessed subjectively, and each item is scored by a binary system. Summed scores range from 0 to 5, where 0 epitomizes robustness, 1 or 2 signifies pre-frail, and scores greater than 2 indicate frailty. Since its creation, it has been successfully applied in community-dwelling elders with excellent prognostic capabilities [19].

The Fried phenotypic model was discarded because our research team deemed gait speed measurements to be potentially difficult to perform and less feasible in a purely orthopedic population. As scaling systems differ, frailty prevalence with both screening instruments was estimated after reclassification as robust or not robust (pre-frail or frail). After obtaining consent, patients were asked to grade their perceived well-being on the Clinical Frailty Scale and to answer all 5 questions of the FRAIL Scale.

### Variables

Patient characteristics were collected before surgery (age, gender, co-morbidity, daily living activities, living status before admission, body mass index (BMI), baseline Hb level), and type of procedure, i.e., total hip or knee arthroplasty (THA or TKA). Hospital mortality, LOS and disposition at discharge were collected by chart review after discharge. Co-morbidity was defined as the co-existence of at least 2 separate chronic illnesses [20]. We quantified the burden of co-morbidity by calculating age-adjusted scores on the Charlson Comorbidity Index [21]. Disability was characterized as difficulty or inability to perform activities essential for independent living, including essential roles, self-care tasks, living independently in a home, and desired activities important to quality of life [19]. We quantified disability with the Katz Index of Independence [22].

### Outcomes

Our primary outcomes were hospital LOS and disposition at discharge. Both have been consistently associated with greater burden and cost to the healthcare system in orthopedic surgery settings [3, 23]. We divided LOS into 3 categories: 1–2 days, 3–5 days, and > 5 days. Short-stay patients (≤2 days) incur the lowest care costs, even when compared to those with LOS of 3–5 days [23], and this can be considered an achievable goal with early, aggressive, in-patient rehabilitation of most of them [24]. LOS of 5 days is often reported as the median value in Canadian patients undergoing TJR [25], and patients with LOS exceeding 5 days incur the highest care costs [23]. Disposition at discharge was characterized by the need to transfer to a rehabilitation center. Secondary outcomes were robust and not robust patient proportions, as defined by both frailty instruments and their agreement.

### Analysis

Frailty prevalence was reported as proportions on both scales. Continuous variables were assessed for normal distribution by the Shapiro-Wilk test, and continuous variables were compared by independent sample t-test (BMI) and the Mann-Whitney test (age, Charlson Comorbidity Index and Hb level). Categorical variables were compared by the Chi-square test or Fisher’s exact test, if absolute patient count was ≤5 for any given category: gender distribution, disposition before surgery, categorized hospital LOS and disposition at discharge. Associations between hospital LOS, discharge to rehabilitation center and frailty were assessed by the Chi-square test or Fisher’s exact test. Unfortunately, our small sample size prevented regression analysis with covariates. Finally, agreement between the 2 frailty scales was evaluated by Cohen’s kappa test. All analyses were performed with SPSS 20.0.

## Results

The baseline characteristics of study subjects are presented in Table 1. Median age of our cohort was 72.0 years (interquartile range (IQR) = 9.0), ranging from 65.0 to 88.0 years, and 65.5% were female patients. More than half of our cohort were living at home with their spouses (57.5%), while 5.6% were living in a residence. The median Charlson Comorbidity Index in our cohort was 4.0 (IQR = 1.0), and 87.4% were fully independent in their daily living activities. TKA represented 51.7% of all procedures, followed by THA and bilateral TJR (39.1% and 9.2%, respectively). The mean time period between pre-operative clinic visit and surgery was 74.3 ± 34.8 days.

**Table 1:** Baseline characteristics of patients stratified by frailty status according to the Clinical Frailty Scale and the FRAIL Scale

	Total patients (*N* = 87)	Clinical Frailty Scale			FRAIL Scale		
		Robust (*n* = 40)	Not robust (*n* = 47)	*p*-value	Robust (*n* = 13)	Not robust (*n* = 74)	*p*-value
Age (median (IQR))	72.0 (9.0	71.0 (8.0)	73.0 (11.0)	0.145	73.0 (6.0)	71.5 (9.0)	0.330
Gender (female) (%)	57 (65.5)	21 (52.5%)	36 (76.6%)	0.018	4 (30.8%)	53 (71.6%)	0.009
Disposition before surgery (%)							
- Home with loved ones	50 (57.5%)	30 (75.5%)	20 (42.6%)	0.02	8 (61.5%)	42 (56.8)	1.00
Charlson comorbidity index (median (IQR))	4.0 (1.0)	4.0 (1.0)	5.0 (1.0)	0.273	5.0 (1.0)	4.0 (1.0)	0.239
Fully independent (%)	76 (87.4)	38 (95%)	38 (82.6%)	0.058	0	11 (14.9%)	0.205
BMI (mean ±SD)	30.5 ± 6.4	28.3 ± 4.7	32.3 ± 7.0	0.002	28.1 ± 3.1	30.9 ± 6.7	0.02
Baseline Hb (median (IQR))	135.0 (16.0)	139.0 (20.0)	134.0 (12.0)	0.109	146.0 (19.0)	134.0 (16.0)	0.004

SD: standard deviation; BMI: body mass index; Hb: hemoglobin; IQR: interquartile range

The Clinical Frailty Scale classified 46.0% of our cohort as robust while the FRAIL Scale grouped 15.0% as robust. Baseline characteristics (gender, disposition before surgery and BMI) differed significantly between robust and not robust patients based on the Clinical Frailty Scale (*p* < 0.05), while fully independent status almost reached statistical significance (*p* = 0.058). Robust and not robust patients did not differ significantly on the FRAIL Scale, except in terms of gender, mean BMI and median baseline Hb level (*p* < 0.05).

Median hospital LOS of our cohort was 5 days (IQR = 3). Eight patients (9.1%) were discharged within 48 h after surgery, 49 patients (56.3%) were discharged between 3 and 5 days, and total LOS was over 5 days in 30 patients (34.4%) (Table 2). No patient died during hospital stay. Based on the Clinical Frailty Scale, 17.5% (*N* = 7) of robust patients had hospital LOS over 5 days compared to 48.9% (*N* = 23) of not robust patients. Hospital LOS distribution differed significantly between robust and not robust patients (*p* = 0.005). When classified according to the FRAIL Scale, hospital LOS distribution did not differ significantly between robust and not robust patients (*p* = 0.700).

**Table 2:** Hospital LOS and discharge to rehabilitation home stratified by frailty status according to the Clinical Frailty Scale and the FRAIL Scale

		Hospital length of stay				Discharge to rehabilitation center		
Frailty scales (N)		1–2 days (8)	3–5 days (49)	> 5 days (30)	*p*-value	Yes (17)	No (70)	*p*-value
Clinical Frailty Scale	Robust	6	27	7	0.005	4	36	0.038
	Not robust	2	22	23		13	34	
FRAIL Scale	Robust	2	7	4	0.700	3	10	0.727
	Not robust	6	42	26		14	60	

A total of 17 patients (19.5%) were discharged to rehabilitation center after surgery. Based on the Clinical Frailty Scale, 10.0% (*N* = 4) of robust patients were transferred to rehabilitation center compared to 27.7% (*N* = 13) of not robust patients (*p* = 0.038). Robust (*N* = 3, 23.1%) and not robust (*N* = 14, 23.3%) patients did not differ significantly on the FRAIL Scale (*p* = 0.727).

Table 3 reports patient classification according to frailty status on the Clinical Frailty Scale and the FRAIL Scale. Since both instruments are not rated by the same scaling system, the results are presented after reclassification into robust, pre-frail and frail patients. The Clinical Frailty Scale classified 40 patients (45.9%) as robust, 43 patients (49.4%) as pre-frail, and 4 patients (4.5%) as frail, while the FRAIL Scale categorized 12 patients (13.7%) as robust, 54 patients (62.0%) as pre-frail, and 20 patients (22.9%) as frail. Both scales agreed on 39 evaluations (38.0%) and disagreed on 48 evaluations (62.0%). Out of the 20 patients considered as frail by the FRAIL Scale, 5 were classified as robust with the Clinical Frailty Scale. Due to the small numbers of patients considered as frail by the latter, both scales were dichotomized into robust versus not robust (sum of pre-frail and frail) (Table 4). Cohen’s kappa agreement was 0.245 (*p* = 0.002) between both scales after dichotomization.

**Table 3:** Comparison of the Clinical Frailty Scale and the FRAIL Scale

	FRAIL Scale			
Clinical Frailty Scale		Robust (0)	Prefrail (1–2)	Frail (3–5)
	Robust (1–3)	11	24	5
	Prefrail (4)	2	27	14
	Frail (5–7)	0	3	1

**Table 4:** Comparison of robustness between the clinical frailty scale and the FRAIL Scale

	FRAIL Scale		
Clinical Frailty Scale		Robust	Not robust
	Robust	11	29
	Not robust	2	45

Cohen’s kappa: 0.245 (*p* = 0.002)

## Discussion

Frailty is progressively acknowledged as a marker of functional decline and a potentially modifiable risk factor for patient improvement. It is even more relevant in peri-operative patients where interventions can be applied before the surgical procedure. In our study, pre-frail and frail patients, as defined by the Clinical Frailty Scale and undergoing TKA or THA, had longer hospital LOS and higher percentage of discharge to rehabilitation center compared to robust patients. These results are consistent with current literature on the general surgical population. Most studies, using Rockwood’s or Fried’s model, have reported associations between increasing frailty and major post-operative outcomes (in-hospital mortality, hospital LOS, post-operative complications, etc.).

In orthopedic surgery, involving the Ontario Healthcare Database, McIsaac et al. [26] found an increase in hospital LOS among frail elderly undergoing THA or TKA based on Johns Hopkins Adjusted Clinical Groups frailty-defining diagnoses indicators (odds ratio: 1.78, confidence interval: 1.74–1.81). The latter model is based on another conceptual underpinning and is also difficult to apply in clinical practice as it relies on variables from multiple datasets. Cooper et al. [27] considered both Fried’s phenotypic model and the Frailty Index in a cohort of 415 orthopedic patients. Both instruments predicted discharge to post-acute institutional care and hospital LOS > 5 days. This discrepancy between results can be explained by the higher prevalence of frailty (35% with Fried’s phenotypic model and 41% with the Frailty Index) in older patients with greater functional decline. They included patients undergoing other types of surgery, such as lumbar and cervical laminectomy.

The FRAIL Scale was not associated with hospital LOS or discharge to rehabilitation center in our cohort. Both scales differed significantly in the percentage of patients classified as robust and not robust, with moderate agreement at best. Part of the difference in performance by both scales can be explained by the modest agreement between them. The 2 instruments are not conceptually identical in their definition of frailty. The Clinical Frailty Scale is derived from the Frailty Index developed by Rockwood and colleagues [17]. It is based on a mathematical model of deficit accumulation and the complex interplay between co-morbidities [15]. The FRAIL Scale is heavily rooted in Fried’s phenotypic model, a syndrome-based definition of weight loss, fatigue and diminished energy expenditure, all related to sarcopenia [28]. Cooper et al. [27] obtained similar results with the phenotypic and deficit accumulation models with moderate agreement (kappa = 0.42). Furthermore, our cohort of orthopedic patients awaiting TJR did not suffer from significant co-morbidities or disabilities, which are all related to frailty. By excluding urgent surgeries, such as hip fracture patients and those deemed too ill for surgery, we further selected a subgroup of healthier and more functional orthopedic patients. In a cohort with higher prevalence of frail patients, the FRAIL Scale may have been associated with clinical outcomes. Also, even with our small sample size, the power of our analyses with the FRAIL scale was 99% for both clinical outcomes. It is unlikely that our results were due to beta errors.

Our study has some limitations. First, it was conducted in a single center with a small sample size, and lacked power to assess other known risk factors and their interactions with frailty status. For example, Hb level was significantly higher in robust patients (with the FRAIL scale) compared to not robust patients, although a multi-centered interventional trial did not show improved outcome with a liberal transfusion strategy in hip surgery patients [29]. Hypoalbuminemia, another risk factor associated with sepsis after TJRs [30], was not captured due to a significant amount of missing data. It is not a mandatory test in our pre-operative clinic. Since it is not, validation is also needed across other cohorts of TJR patients. Second, these results cannot be applied to other surgical specialties, since frailty prevalence might differ from orthopedic patients, and it is still unclear which frailty instrument is better suited for a specific surgical subgroup. Third, as mentioned, we excluded urgent surgical procedures as the prevalence of frailty is usually higher in this group. In a previous study evaluating frailty with the Frailty Index in hip fracture patients, the prevalence of being very frail on admission was 36%. Therefore, our findings may not apply to them.

As for our study’s strengths, the recruitment process did not delay surgery and usual care, and was easily implemented in routine assessment due to the simplicity of both selected scales. Our results agree with the existing literature and, to our knowledge, it is the first time that the Clinical Frailty Scale and the FRAIL Scale have been applied in a TJR population.

## Conclusion

In conclusion, the Clinical Frailty Scale was, while the FRAIL scale was not, significantly associated with hospital LOS and discharge to rehabilitation center in our cohort of TJR patients. Whether either instrument can be generalized to other surgical patients and used to target vulnerable elderly patients for pre-operative intervention needs to be investigated.

## Abbreviations

BMI: Body mass index
Hb: Hemoglobin
IQR: Interquartile range
LOS: Length of stay
THA: Total hip arthroplasty
TJR: Total joint replacement
TKA: Total knee arthroplasty
